# The effects of age on dyspnea and respiratory mechanical and neural responses to exercise in healthy men

**DOI:** 10.14814/phy2.15794

**Published:** 2023-08-21

**Authors:** William MacAskill, Ben Hoffman, Michael A. Johnson, Graham R. Sharpe, Joshua Rands, Shoena E. Wotherspoon, Yaroslav Gevorkov, Tracy L. Kolbe‐Alexander, Dean E. Mills

**Affiliations:** ^1^ School of Health and Medical Sciences University of Southern Queensland Ipswich Queensland Australia; ^2^ Respiratory and Exercise Physiology Research Group, School of Health and Wellbeing University of Southern Queensland Ipswich Queensland Australia; ^3^ Centre for Health Research Institute for Resilient Regions, University of Southern Queensland Ipswich Queensland Australia; ^4^ Rural Clinical School Griffith University Toowoomba Queensland Australia; ^5^ Exercise and Health Research Group, Sport, Health and Performance Enhancement (SHAPE) Research Centre, School of Science and Technology Nottingham Trent University Nottinghamshire UK; ^6^ Darling Downs Health, Queensland Health Queensland Australia; ^7^ Institute of Vision Systems, Hamburg University of Technology Hamburg Germany; ^8^ UCT Research Centre for Health through Physical Activity, Lifestyle and Sport (HPALS), Division of Research Unit for Exercise Science and Sports Medicine, Faculty of Health Sciences University of Cape Town Cape Town South Africa

**Keywords:** aging, dyspnea, exercise, mechanical, neural

## Abstract

The respiratory muscle pressure generation and inspiratory and expiratory neuromuscular recruitment patterns in younger and older men were compared during exercise, alongside descriptors of dyspnea. Healthy younger (*n* = 8, 28 ± 5 years) and older (*n* = 8, 68 ± 4 years) men completed a maximal incremental cycling test. Esophageal, gastric (*P*_ga_) and transdiaphragmatic pressures, and electromyography (EMG) of the crural diaphragm were measured using a micro‐transducer and EMG catheter. EMG of the parasternal intercostals, sternocleidomastoids, and rectus abdominis were measured using skin surface electrodes. After the exercise test, participants completed a questionnaire to evaluate descriptors of dyspnea. *P*_ga_ at end‐expiration, *P*_ga_ expiratory tidal swings, and the gastric pressure–time product (PTP_ga_) at absolute and relative minute ventilation were higher (*p* < 0.05) for older compared to younger men. There were no differences in EMG responses between older and younger men. Younger men were more likely to report shallow breathing (*p* = 0.005) than older men. Our findings showed younger and older men had similar respiratory neuromuscular activation patterns and reported different dyspnea descriptors, and that older men had greater expiratory muscle pressure generation during exercise. Greater expiratory muscle pressures in older men may be due to compensatory mechanisms designed to offset increasing airway resistance due to aging. These results may have implications for exercise‐induced expiratory muscle fatigue in older men.

## Graphical abstract

In this paper, we report on respiratory muscle pressure generation and inspiratory and expiratory neuromuscular recruitment patterns during incremental exercise in older compared to younger men. Our findings showed younger and older men had similar respiratory neuromuscular activation patterns, reported different dyspnea descriptors, and that older men had greater expiratory muscle pressure generation during exercise. These results may be due to compensatory mechanisms designed to offset increasing airway resistance due to aging and could therefore have implications for exercise‐induced expiratory muscle fatigue in older men.

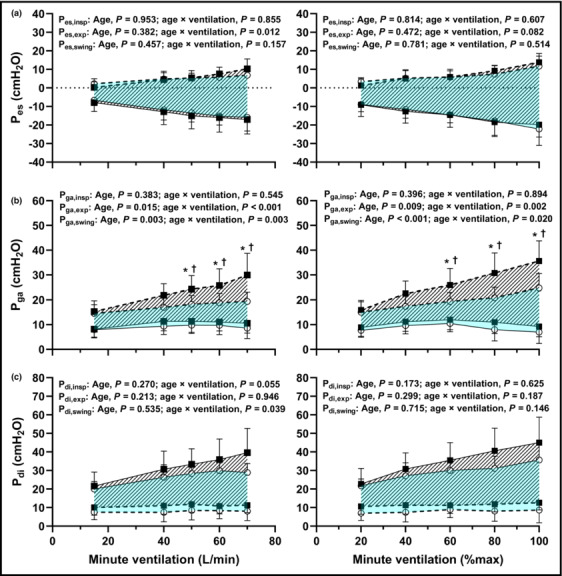

## 1 INTRODUCTION

Healthy aging results in progressive changes to the lungs, airways, chest wall, and respiratory muscles, leading to a decline in respiratory structure and function (Janssens et al., 1999; Lalley, 2013; Meiners et al., 2015; Roman et al., 2016). The changes that occur during healthy aging include reductions in chest wall compliance due to structural changes in the thoracic cage (Estenne et al., 1985; Mittman et al., 1965) including kyphosis, calcification of the costal cartilage, narrowing of intervertebral disc spaces, and osteoporosis (Holcombe et al., 2017). Additionally, lung compliance progressively increases (Turner et al., 1968), possibly due to deterioration in the spatial arrangement and/or cross linking of the elastic fiber network (Fukuchi, 2009). Respiratory muscle function also declines with age, due to the atrophy and denervation of type II muscle fibers (Elliott et al., 2016; Ticinesi et al., 2017). There is evidence that this also occurs within the diaphragm due to a loss of larger phrenic motoneurons (Fogarty et al., 2018; Prakash & Sieck, 1998).

Inspiratory muscle pressure generation and the resistive and elastic components of the work of breathing (WOB) are greater for older compared to younger adults during maximal incremental exercise (Molgat‐Seon et al., 2018b, Molgat‐Seon et al., 2019, Weavil et al., 2022, Molgat‐Seon et al., 2018a). Molgat‐Seon et al. (2018b) reported that inspiratory neuromuscular recruitment patterns during maximal incremental exercise are different between healthy younger and older adults. Older adults had greater esophageal and transdiaphragmatic pressure–time products (PTP_es_ and PTP_di_) at absolute minute ventilations ($\dot{V}$_E_) compared to younger adults (Molgat‐Seon et al., 2018b). Older adults also had greater recruitment of primary and accessory inspiratory muscles, evidenced by their higher amplitudes for crural diaphragm electromyography (EMG_di_), scalene EMG and sternocleidomastoids EMG (EMG_scm_) at absolute ${\dot{V}}_{E}$ (Molgat‐Seon et al., 2018b). At relative ${\dot{V}}_{E}$, EMG_scm_ was also higher for older compared to younger adults (Molgat‐Seon et al., 2018b). While the work of Molgat‐Seon et al. (2018b) provides valuable insight into the differences between younger and older adults in inspiratory muscle pressure generation and neuromuscular recruitment patterns during exercise, they did not report on the contributions of the expiratory muscles. With advancing age, the muscle mass of the rectus abdominis declines (Abe et al., 2011; Kubo, 1994) and abdominal wall compliance is reduced (Estenne et al., 1985). Therefore, compared to younger men, greater abdominal muscle recruitment may be expected in older men to support the inspiratory role of the abdominal musculature, and to compensate for their greater expiratory flow limitation and potentially higher expiratory WOB during exercise (Smith et al., 2018; Weavil et al., 2022).

Dyspnea is defined as “a subjective experience of breathing discomfort that consists of qualitatively distinct sensations that vary in intensity” (American Thoracic Society, 1999, Parshall et al., 2012). Higher ratings of perceived exertion (RPE) for dyspnea have been reported in older compared to younger men (Ofir et al., 2008), although others report no difference (Faisal et al., 2015). Dyspnea is influenced by a wide variety of stimuli and sensory receptors which contributes to the challenges in fully understanding its mechanistic causes (Laviolette & Laveneziana, 2014; Parshall et al., 2012). Ofir et al. (2008) compared descriptors of dyspnea between younger and older men and women following maximal incremental treadmill exercise and found that shallow breathing was reported by a greater proportion of older men and women. Breathing discomfort was also higher for older than younger women. Older men only reported greater breathing discomfort than young men at higher relative oxygen uptake (≥25 mL/kg/min) (Ofir et al., 2008). Descriptors of dyspnea may provide a more complete understanding of its subjective experience and may assist in developing a more comprehensive understanding of dyspnea (Lansing et al., 2009). Due to age‐related changes in respiratory physiology, older men may report a different affective experience of dyspnea during exercise. No published literature has measured detailed respiratory muscle pressure generation and neuromuscular recruitment patterns, including expiratory measurements, alongside descriptors of dyspnea in younger and older men during incremental exercise.

The aim of this study was to compare respiratory muscle pressure generation, inspiratory and expiratory neuromuscular recruitment patterns, and descriptors of dyspnea, in younger and older men during exercise. We hypothesized that older men would have greater expiratory muscle recruitment and expiratory muscle pressure generation during exercise and select descriptors of dyspnea consistent with an increased respiratory muscle pressure generation following exercise.

## 2 METHODS

### 2.1 Participants

Healthy younger (*n* = 8) and older (*n* = 8) men participated in this study (Table 1). The exclusion criteria were current cigarette smokers (two older adults quit smoking more than 10 years ago); history or current symptoms of cardiopulmonary disease; prescribed medications affecting the cardiopulmonary system; contraindications to exercise testing; and a body mass index of below 18.5 or above 30 kg/m^2^. Participants were aged between 18–35 (younger) or 65–80 (older) years and recreationally active. The definition of a recreationally active participant for this study was participation in nonprofessional sport one to three times per week. The study was approved by the University of Southern Queensland's Human Research Ethics Committee and all procedures conformed to the standards set by the Declaration of Helsinki, except for registration in a database. The trial was registered with the Australian and New Zealand Clinical Trial Registry (ACTRN 376141). All participants provided written, informed consent prior to participation.

**TABLE 1:** Participant characteristics and pulmonary function for the younger and older men.

	Younger men	Older men	*p*‐value
Age, years	28 ± 5	68 ± 4	**<0.001**
Height, cm	179 ± 6	180 ± 7	0.853
Body mass, kg	83 ± 9	86 ± 14	0.707
BMI, kg/m^2^	26 ± 3	26 ± 3	0.787
FVC, L	5.2 ± 0.8	4.5 ± 1.1	0.153
FVC, %predicted	98 ± 12	102 ± 16	0.589
FEV_1_, L	4.1 ± 0.6	3.3 ± 0.5	**0.007**
FEV_1_, %predicted	95 ± 13	99 ± 14	0.579
FEV_1_/FVC, %	81 ± 6	75 ± 10	0.136
FEV_1_/FVC, %predicted	97 ± 8	99 ± 13	0.856
P_es,max_, cmH_2_O	−131 ± 52	−112 ± 39	0.432
P_ga,max_, cmH_2_O	138 ± 71	132 ± 45	0.853
P_di,max_, cmH_2_O	71 ± 7	79 ± 11	0.096

Values are mean ± SD.

Abbreviations: BMI, body mass index; FVC, forced vital capacity; FEV_1_, forced expiratory volume in 1 s; P_es,max_, maximum esophageal pressure; P_ga,max_, maximum gastric pressure; P_di,max_, maximum transdiaphragmatic pressure. P_es,max_ and P_di,max_ were collected during a maximal inspiratory pressure maneuver. P_ga,max_ was collected during a maximal expiratory pressure maneuver.

### 2.2 Experimental design

The study utilized a cross‐sectional design. Each participant visited the laboratory on two occasions, separated by a minimum of 48 h and a maximum of 1 week. Visit 1 included screening for the eligibility criteria, anthropometric and pulmonary function assessment, and familiarization with all the experimental procedures. Visit 2 included a maximal incremental cycling test with measurements of respiratory muscle pressures and EMG, and ventilatory, cardiovascular and perceptual responses to exercise. Participants were instructed to abstain from food (4 h), caffeine (12 h), and strenuous exercise (48 h) before testing.

### 2.3 Anthropometrical measures and pulmonary function

Height and body mass were recorded using a wall mounted electronic stadiometer (Seca 213; Seca) and an electronic scale (Tanita BC‐541), respectively. Body mass index was then calculated as body mass divided by height in meters squared. Pulmonary function was assessed using a spirometer (Vmax® Encore PFT system; Vyaire Medical) according to published guidelines (Miller et al., 2005). Pulmonary function measurements were expressed as absolute values and as percentages of predicted values (Quanjer et al., 2012).

### 2.4 Maximal incremental cycling

Maximal incremental cycling was conducted on an electronically braked cycle ergometer (Corival CPET; Lode) automatically controlled by software (SentrySuite; Vyaire Medical). The protocol consisted of a baseline resting period of 5 min (while participants were seated on the ergometer) followed by 1 min of unloaded cycling. The initial work rate was individualized for each participant, with participants beginning at either 40, 60, or 80 W, depending upon their age, and self‐reported exercise training history. Work rate was increased by 20 W every 2 min. Participants maintained a constant self‐selected cadence above 60 revs/min. Exercise ceased at the limit of tolerance or when cycling cadence could not be maintained above 60 revs/min.

### 2.5 Respiratory pressures and electromyography

Esophageal pressure (*P*_es_), gastric pressure (*P*_ga_), transdiaphragmatic pressure (*P*_di_), and EMG_di_ were measured in real time using a micro‐transducer and EMG esophageal catheter as previously described (MacAskill et al., 2021). The catheter housed two pressure transducers (~5 × 2 mm), separated by 22.8 cm, which were constructed using half bridge thin film resistive strain gauge sensors coated with a silicone elastomer with frequency responses of 10–20 kHz. The catheter comprised a 100 cm silicon shaft (2.7 mm diameter) containing nine silver electrodes spaced 1 mm apart and the pressure transducers were positioned proximally and distally to the electrodes. The nine electrodes formed five pairs (i.e., electrodes 1 & 5; 2 & 6; 3 & 7; 4 & 8; 5 & 9), with each pair recording a difference in electrical potential across the crural diaphragm. The highest mean EMG_di_ recorded from any of the catheters five EMG pairs was utilized for analysis (Dacha et al., 2019).

Prior to instrumentation, the catheter was soaked in water for 1 h as per manufacturer's instructions to reduce baseline drift. The micro‐transducer catheter was then placed inside a small section of airtight plastic tubing and calibrated by injecting or withdrawing air, via a three‐way open valve connected to a glass syringe and a handheld respiratory pressure meter (Micro RPM; Vyaire Medical). *P*_es_ and *P*_ga_ were calibrated from −100 cmH_2_O to +100 cmH_2_O. Catheter placement was preceded by the administration of 91% aerosolized lidocaine hydrochloride and 9% phenylephrine hydrochloride (Co‐Phenylcaine Forte Spray; Almed). The catheter was passed peri‐nasally into the stomach until a negative deflection in *P*_es_ and a positive deflection in *P*_ga_ were observed during repeated sniffs. Further sniffs were performed (to monitor *P*_es_ and *P*_ga_ inflection) while the catheter position was optimized to record maximal EMG_di_ on the superior and inferior electrode pairs. An occlusion test was then performed to confirm the catheters location in the esophagus before securing the catheter in position (Baydur et al., 1982).

*P*_di_ was calculated by subtracting *P*_es_ from *P*_ga_. *P*_es_, *P*_ga_ and *P*_di_ were recorded throughout exercise. Additionally, these signals were integrated for active pressures over each breath's periods of inspiration (*P*_di_ and *P*_es_) and expiration (*P*_ga_), then multiplied by breathing frequency to calculate PTP_di_, PTP_es_, and gastric pressure time products (PTP_ga_). Pressures were normalized using the maximum pressure obtained from any forced vital (for *P*_ga_) or inspiratory (for *P*_di_ and *P*_es_) capacity maneuvers performed during pulmonary function assessment or exercise testing.

Skin‐surface parasternal EMG (EMG_para_), EMG_scm_, and EMG rectus abdominis (EMG_ra_) were assessed using pairs of bipolar skin‐surface electrodes (Ambu WhiteSensor 40,713). The electrodes were placed on the right side of the torso after shaving (if required), cleaning and abrading the skin with skin preparation gel (NuPrep; Weaver and Company) and alcohol wipes. The electrodes were 2 cm in diameter and an interelectrode distance of 2 cm was utilized. Surface electrodes for EMG_para_ and EMG_scm_ were placed as described previously (Ramsook et al., 2016). EMG_para_ were placed in the space of the second rib roughly 3 cm lateral to the sternum, and for EMG_scm_ at the midpoint along the longitudinal axis of the sternocleidomastoid muscle between the mastoid process and the medial clavicle. EMG_ra_ were placed 2 cm superior and 2–4 cm lateral to the umbilicus (Fuller et al., 1996; Ng et al., 1998). Optimal electrode positioning was determined by utilizing maximal inspiratory (EMG_para_ and EMG_scm_) and expiratory (EMG_ra_) mouth pressure measurements to evoke EMG responses. EMG data were transformed into root mean square (RMS) with a time constant of 0.05 s. EMG RMS values were manually selected between the QRS complexes of the electrocardiogram (ECG) to limit the effect of cardiac artifacts on respiratory muscle recruitment (Guenette et al., 2014; Jolley et al., 2015; Schaeffer et al., 2014). EMG was normalized using the maximum EMG obtained from any forced vital (EMG_ra_) or inspiratory (EMG_di_, EMG_para_ and EMG_scm_) capacity maneuvers performed during pulmonary function assessment or exercise testing.

### 2.6 Metabolic, Ventilatory, and cardiorespiratory measurements

Participants wore a facemask (Model 7940; Hans Rudolph) which was tightly fitted to minimize leaks and connected to a turbine flow sensor (Digital volume transducer; Vyaire Medical) that was calibrated using a 3 L syringe. Pulmonary gas exchange was measured breath by breath using a metabolic cart (Vmax® Encore PFT system; Vyaire Medical). As the metabolic cart could not be integrated with the data acquisition unit, flow measurements were additionally recorded with a heated calibrated pneumotachograph (Model 3813; Hans Rudolph) and were time aligned with pressure and EMG data. The pneumotachograph was attached distally to the flow sensor from the metabolic cart. This assembly (facemask, pneumotach, and flow sensor) has an estimated total dead space of 144 mL. Cardiac frequency and ECG responses were monitored continuously during testing using a 12‐lead ECG (PC‐ECG 1200; Norav). Arterial oxygen saturation was estimated using infrared fingertip pulse oximetry (Model 8600; Nonin). The maximal work rate was calculated as the work rate of the penultimate stage plus the fraction of work completed in the final stage. The highest oxygen uptake recorded during any 30 s period defined maximal oxygen uptake ($\dot{V}$O_2max_). Predicted $\dot{V}$O_2max_ was determined using a reference equation for healthy men undertaking cycling exercise (de Souza e Silva et al., 2018). Predicted maximal cardiac frequency was determined using published formulas for healthy men free of cardiovascular disease (Ozemek et al., 2017).

### 2.7 Operational lung volumes

Volume was obtained by numerical integration of the flow signal. Operational lung volumes were quantified by measuring inspiratory capacity (IC) relative to forced vital capacity (FVC). During the resting stage, participants performed FVC and IC maneuvers in duplicate. Participants performed further IC maneuvers in duplicate during the exercise testing within the final 1 min of each incremental stage. Strong verbal encouragement was given during each maximal inspiratory effort maneuver.

### 2.8 Perceptual responses

Intensities of breathing (dyspnea) and leg discomfort were rated using Borg's CR‐10 RPE scale (Borg, 1982). Breathing discomfort was defined as “a feeling of labored or difficult breathing,” and leg discomfort as “a feeling of fatigue in the leg muscles.” In this scale 0 represents “no discomfort” while 10 represents “maximum imaginable discomfort” (Borg, 1982). Reporting was completed at rest, at the end of each stage, and immediately following the cessation of exercise. After the exercise test, participants completed a questionnaire to evaluate descriptors of dyspnea. This questionnaire was a modified version of those previously utilized by Simon et al. (1990) and Cory et al. (2015). Participants were asked to indicate whether each descriptor was applicable or not and their responses were recorded.

### 2.9 Data capture and analysis of exercise end points

Raw pressure and EMG data were amplified with a Quad Bridge Amp FE224 and Octal Bio Amp FE238 (AD Instruments), respectively. Pressure data were low‐pass filtered at 1 kHz and EMG data were high‐pass filtered at 80 Hz. Pressure, EMG, and flow data were sampled continuously at 10 kHz using a 16‐channel analog‐to‐digital data acquisition system (PowerLab 16/35; AD Instruments) and recorded using LabChart v8.1.2 software (ADInstruments).

Nonphysiological pressure, EMG, and flow data that resulted from swallowing, coughing, and breath holding were identified by visual inspection and removed. All physiological variables collected during the cycle ergometer test were averaged in 30 s epochs. Data were collected in the final 1 min of each 2 min stage and used for statistical analysis. During this time, participants were asked to look forward, minimize any head or neck movement, keep a loose grip on the handlebars, and to avoid talking or swallowing. Five representative and consecutive breaths were selected from the final 30 s of each 2 min stage for analysis (Dacha et al., 2019).

The schedule of data collection in each 2 min exercise stage was as follows: 0–60 s, exercise only; 61–90 s, IC maneuvers; and 91–120 s, once breathing pattern returned to baseline perceptual responses and representative breaths were collected.

### 2.10 Statistical analyses

Statistical analyses were performed using SPSS for Windows (IBM). An initial power calculation was performed based on differences in *P*_ga_ swings during exercise between younger and older men. Power analysis indicated that a sample size of 10 (5 younger and 5 older men) would be required to detect differences in *P*_ga_ swings (alpha = 0.05, power = 0.8) and provide a large effect size (*dz* = 2.1).

Normality was assessed using a Shapiro–Wilk test. Comparisons between younger and older men for characteristics, pulmonary function and peak metabolic, ventilatory and cardiorespiratory responses were determined using an independent *t*‐test and a Mann–Whitney *U* ‐test for parametric and nonparametric data, respectively. The older and younger men were compared at rest and at matched ${\dot{V}}_{E}$ of ~40, 50, 60, and 70 L/min (31–45, 46–55, 56–65, 66–75 L/min), as 70 L/min was the highest ${\dot{V}}_{E}$ reached by all participants. The older and younger men were also compared at matched relative ${\dot{V}}_{E}$ of ~20, 40, 60, 80 and 100% of maximum (11–30, 31–50, 51–70, 71–99, 100% maximum). Interpolation was used if necessary to match participant ${\dot{V}}_{E}$.

Between‐group differences were analyzed with a two‐way analysis of variance to determine the effects of “age” (younger vs. older) and “${\dot{V}}_{E}$” (absolute ${\dot{V}}_{E}$: rest and at ~40, 50, 60, and 70 L/min; or relative ${\dot{V}}_{E}$: ~20, 40, 60, 80, and 100% maximum). Significant main effects of age and age × ventilation interaction effects were followed by planned pairwise comparisons between ages using the Bonferroni post hoc analysis. Comparisons between younger and older participants for categorical descriptors of dyspnea were made using Fisher's Exact test. Statistical significance was set at *p* ≤ 0.05. Values are expressed as means ± SD.

## 3 RESULTS

### 3.1 Participant characteristics and pulmonary function

There were no differences in participant characteristics and pulmonary function between younger and older men, apart from forced expiratory volume in 1 s (FEV_1_) which was lower for older men (Table 1).

### 3.2 Metabolic, Ventilatory, and cardiorespiratory responses

The metabolic, ventilatory and cardiorespiratory responses for the younger and older men at peak exercise are shown in Table 2. At peak exercise, the percentage of predicted $\dot{V}$O_2max_ was higher, whereas carbon dioxide output, respiratory exchange ratio, minute ventilation, ${\dot{V}}_{E}$/$\dot{V}$O_2_ and cardiac frequency were lower, in older compared to younger men. Significant age × ventilation interaction effects were not observed at either absolute or relative ${\dot{V}}_{E}$.

**TABLE 2:** Metabolic, ventilatory, and cardiorespiratory responses for younger and older men at peak exercise.

	Younger men	Older men	*p*‐value
W_peak_, W	212 ± 52	206 ± 37	0.816
$\dot{V}$O_2_, L/min	3.22 ± 0.64	2.55 ± 1.15	0.174
$\dot{V}$O_2_, mL/kg/min	37.3 ± 5.6	33.7 ± 8.5	0.330
$\dot{V}$O_2_, %predicted	87.8 ± 17.3	115.2 ± 26.8	**0.030**
$\dot{V}$CO_2_, L/min	3.56 ± 0.70	2.51 ± 1.05	**0.033**
RER	1.18 ± 0.08	1.02 ± 0.16	**0.024**
PETO_2_, mmHg	119 ± 5	117 ± 7	0.355
PETCO_2_, mmHg	33.7 ± 3.4	31.6 ± 6.2	0.408
${\dot{V}}_{E}$, L/min	130 ± 24	94 ± 37	**0.034**
${\dot{V}}_{E}$/$\dot{V}$O_2_	41.3 ± 1.6	37.0 ± 4.6	**0.047**
${\dot{V}}_{E}$/$\dot{V}$CO_2_	34.7 ± 2.7	38.2 ± 7.3	0.312
F_b_, breaths/min	50 ± 10	38 ± 13	0.119
V_T_, L	2.76 ± 0.55	2.57 ± 0.80	0.594
V_T_, % FVC	53 ± 7	64 ± 13	0.065
T_I_, s	0.61 ± 0.11	0.69 ± 0.11	0.180
T_TOT_, s	1.24 ± 0.22	1.46 ± 0.23	0.072
T_I_/T_TOT_	0.48 ± 0.01	0.47 ± 0.02	0.656
IC, L	3.45 ± 0.82	3.24 ± 0.82	0.660
F_c_, beats/min	179 ± 14	149 ± 13	**0.001**
F_c_, %predicted	92 ± 7	93 ± 8	0.098
SpO_2_, %	95 ± 2	95 ± 2	0.611

Values are mean ± SD.

Abbreviations: W_peak_, peak work rate; $\dot{V}$O_2_, oxygen uptake; $\dot{V}$CO_2_, carbon dioxide output; RER, respiratory exchange ratio; PETO_2_, end tidal oxygen pressure; PETCO_2_, end tidal carbon dioxide pressure; ${\dot{V}}_{E}$, minute ventilation; F_b_, breathing frequency; V_T_, tidal volume; T_I_, inspiratory time, T_TOT_, total breath time; T_I_/T_TOT_, duty cycle; IC, inspiratory capacity; FVC, forced vital capacity; F_c_, cardiac frequency; SpO_2_, estimated arterial oxygen saturation.

### 3.3 Respiratory pressure generation

**FIGURE 1:**
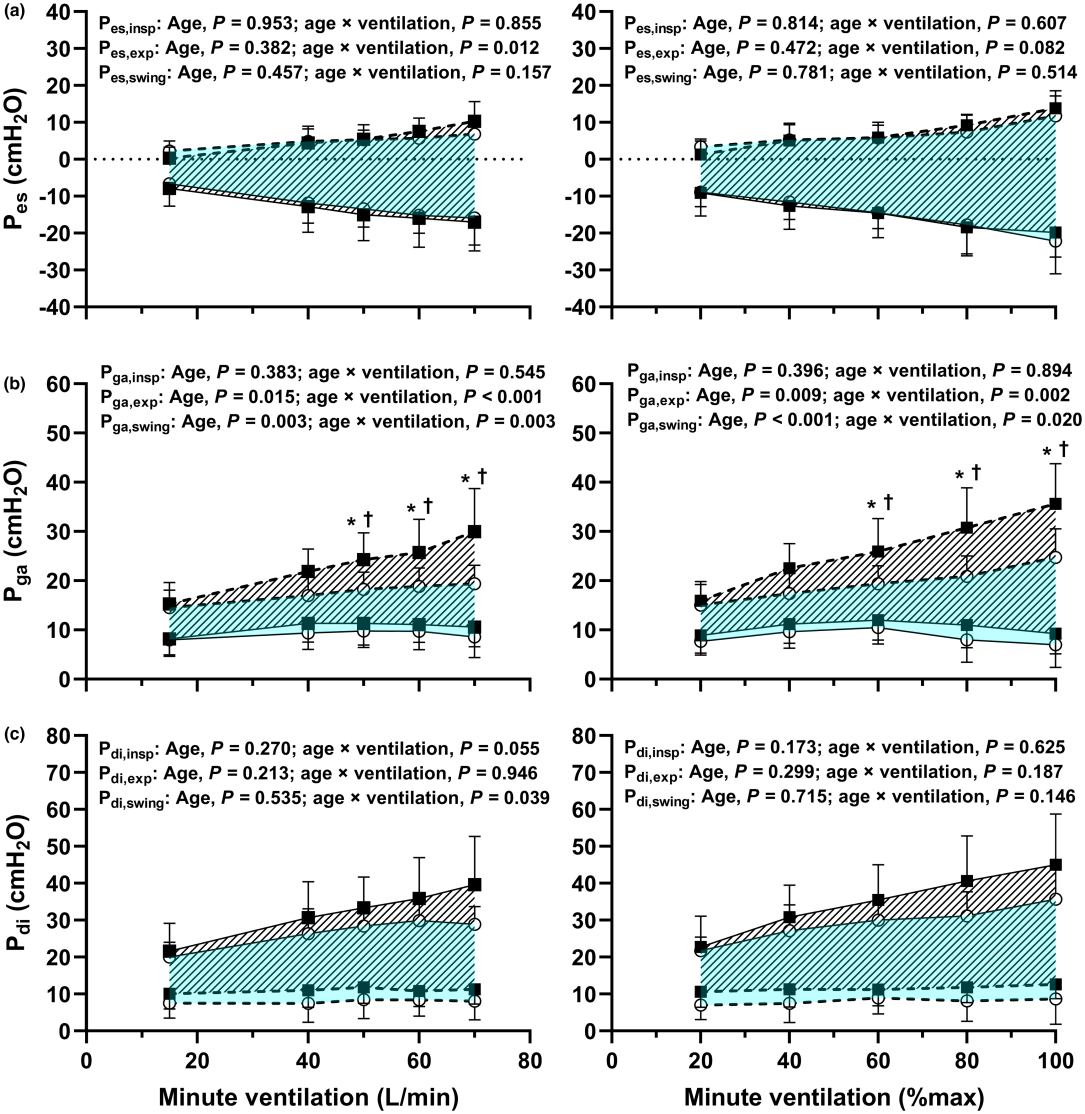
Esophageal (*P*_es_; a), gastric (*P*_ga_; b) and transdiaphragmatic (*P*_di_; c) pressures at rest and during incremental exercise. End‐inspiration (*P*_insp_, solid lines) and expiration (*P*_exp_, dashed lines) are shown for absolute (left hand panels) and relative (right hand panels) minute ventilation for younger (open circles) and older men (filled boxes). Tidal pressure swings (*P*_swing_) are represented by the pressure difference between points of end‐inspiration and end‐expiration and are shown for younger (blue fill) and older (diagonal fill) men. Data are presented as mean ± SD. Main effects of age and age × ventilation interaction effects are provided in each panel. Significant difference in pressures at (*) end‐expiration and (†) tidal pressure swings between younger and older men (*p* < 0.05).

**FIGURE 2:**
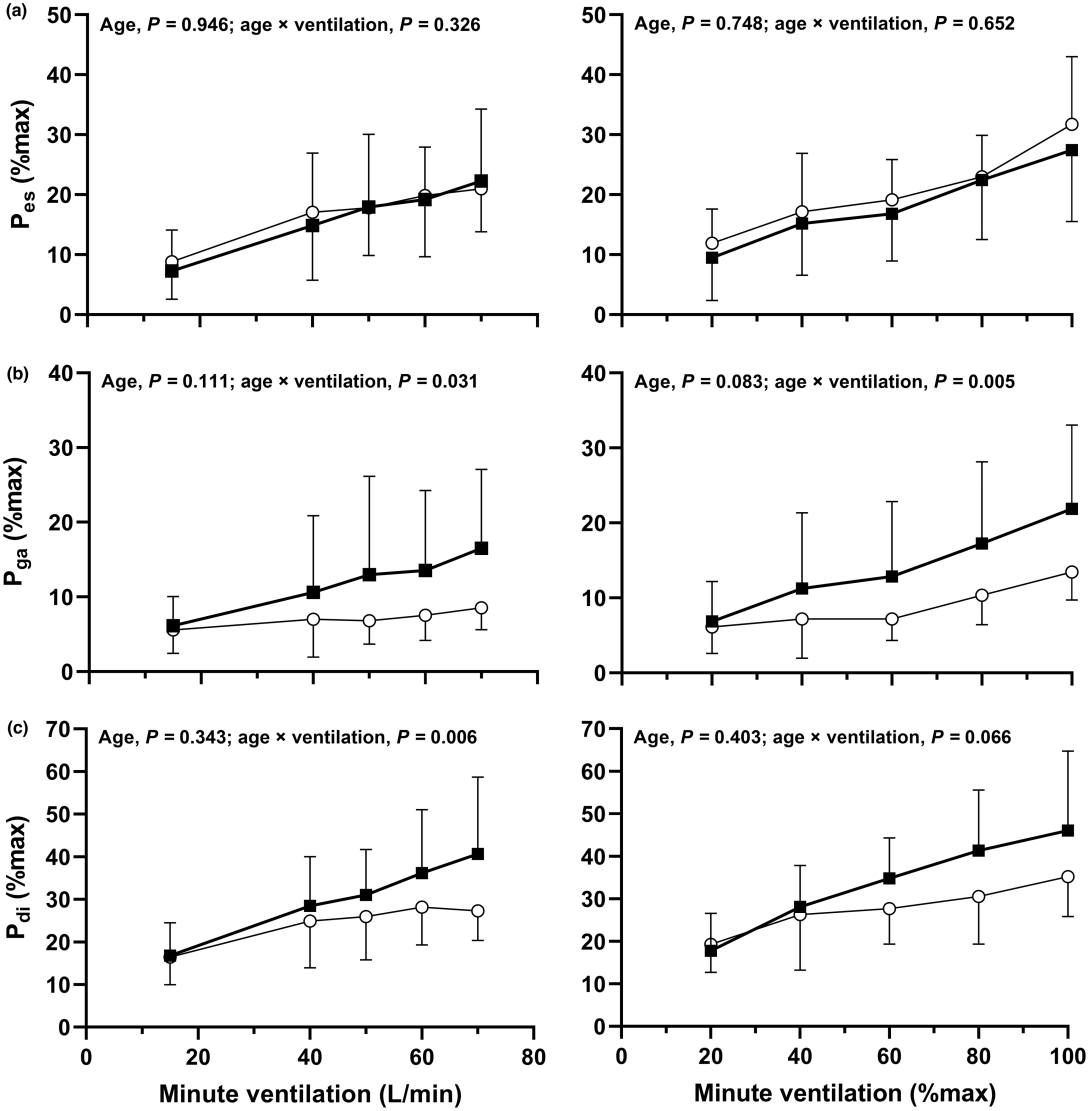
Esophageal (*P*_es_; a), gastric (*P*_ga_; b), and transdiaphragmatic (*P*_di_; c) pressure swings as a percentage of maximum at rest and during maximal incremental exercise. Relative pressures are expressed relative to maximal *P*_ga_. Responses at absolute (left hand panels) and relative (right hand panels) minute ventilation are shown for younger (open circles) and older men (filled boxes). Data are presented as mean ± SD. Main effects of age and age × ventilation interaction effects are provided in each panel.

**FIGURE 3:**
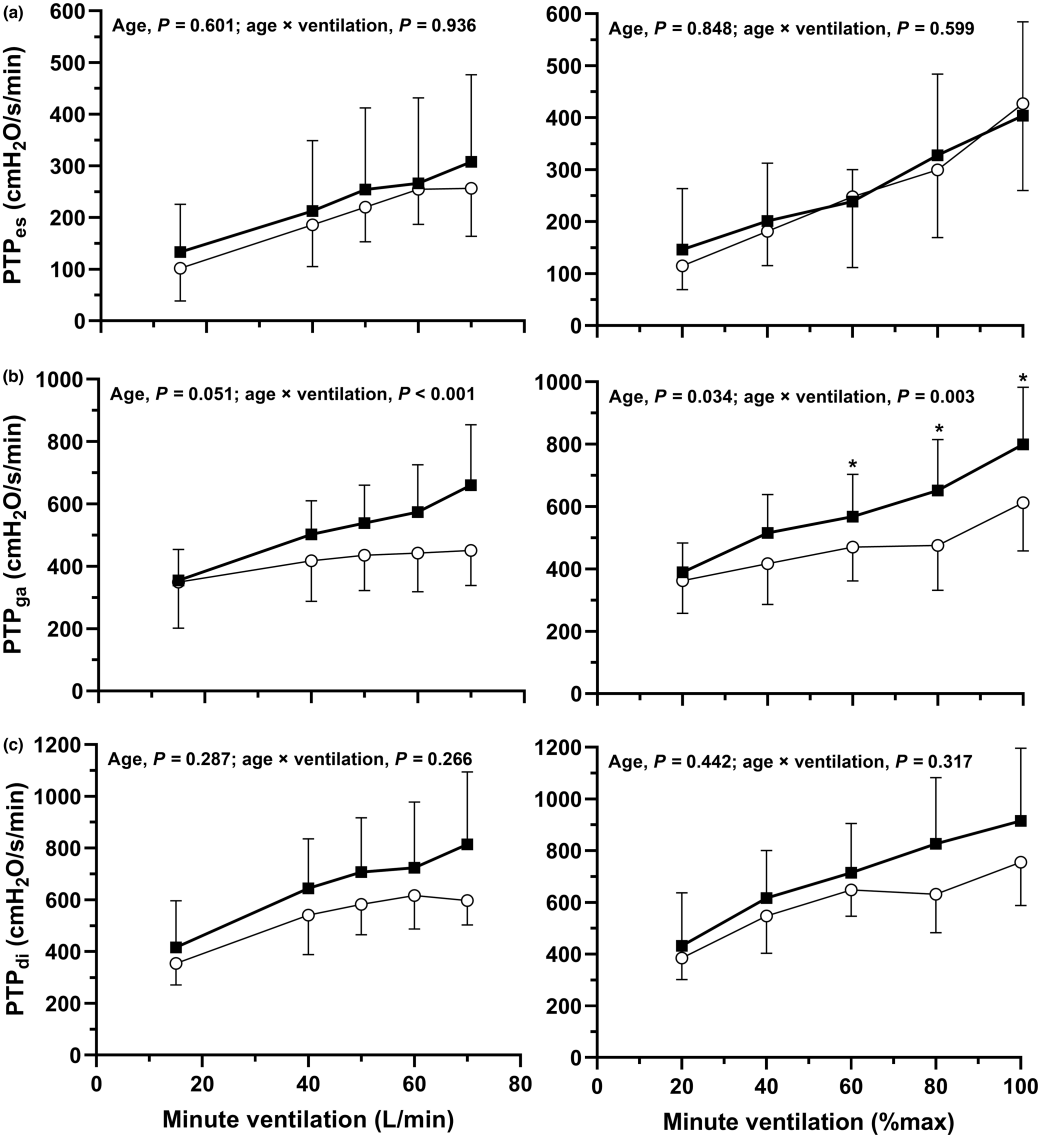
Esophageal (PTP_es_; a), gastric (PTP_ga_; b), and transdiaphragmatic (PTP_di_; c) pressure–time products at rest and during maximal incremental exercise. Responses at absolute (left hand panels) and relative (right hand panels) minute ventilation are shown for younger (open circles) and older men (filled boxes). Data are presented as mean ± SD. Main effects of age and age × ventilation interaction effects are provided in each panel. *Significant difference between younger and older men (*p* < 0.05).

Absolute *P*_es_, *P*_ga_, and *P*_di_ and pressure swings are shown in Figure 1. Pressures as a percentage of maximum are shown in Figure 2. PTPs are shown in Figure 3. Main effects of age and age × ventilation interaction effects are shown in the figures. All measurements increased during exercise (main effect of absolute and relative ${\dot{V}}_{E}$, *p* < 0.01), except for *P*_di_ at end‐expiration (absolute ${\dot{V}}_{E}$, *P* = 0.431; relative ${\dot{V}}_{E}$, *p* = 0.354).

Main effects of age were observed for *P*_ga_ at end‐expiration and *P*_ga_ swing (at absolute and relative ${\dot{V}}_{E}$) and PTP_ga_ (at relative ${\dot{V}}_{E}$). This effect showed that older men's *P*_ga_ at end‐expiration was higher, and *P*_ga_ swings larger (Figure 1). Age × ventilation interactions were observed for *P*_ga_ at end‐expiration and PTP_ga_ (at relative and absolute ${\dot{V}}_{E}$) and for *P*_ga_ swing (at relative ${\dot{V}}_{E}$). Pairwise differences in *P*_ga_ at end‐expiration and *P*_ga_ swings were apparent from ${\dot{V}}_{E}$ = 50 L/min and ${\dot{V}}_{E}$ = 60% until the end of the exercise test (*p* < 0.05). Pairwise differences in PTP_ga_ occurred from ${\dot{V}}_{E}$ = 80% until the end of the exercise test (*p* < 0.05). When *P*_ga_ tidal swings were expressed as a percentage of maximum, an age × ventilation effect was observed (at absolute and relative ${\dot{V}}_{E}$), indicating greater swings in older men (Figure 2). Age × ventilation interactions were also observed at absolute ${\dot{V}}_{E}$ for *P*_es_ at end‐expiration, *P*_di_ swing, and *P*_di_ swing as a percentage of maximum.

### 3.4 Respiratory muscle electromyography

**FIGURE 4:**
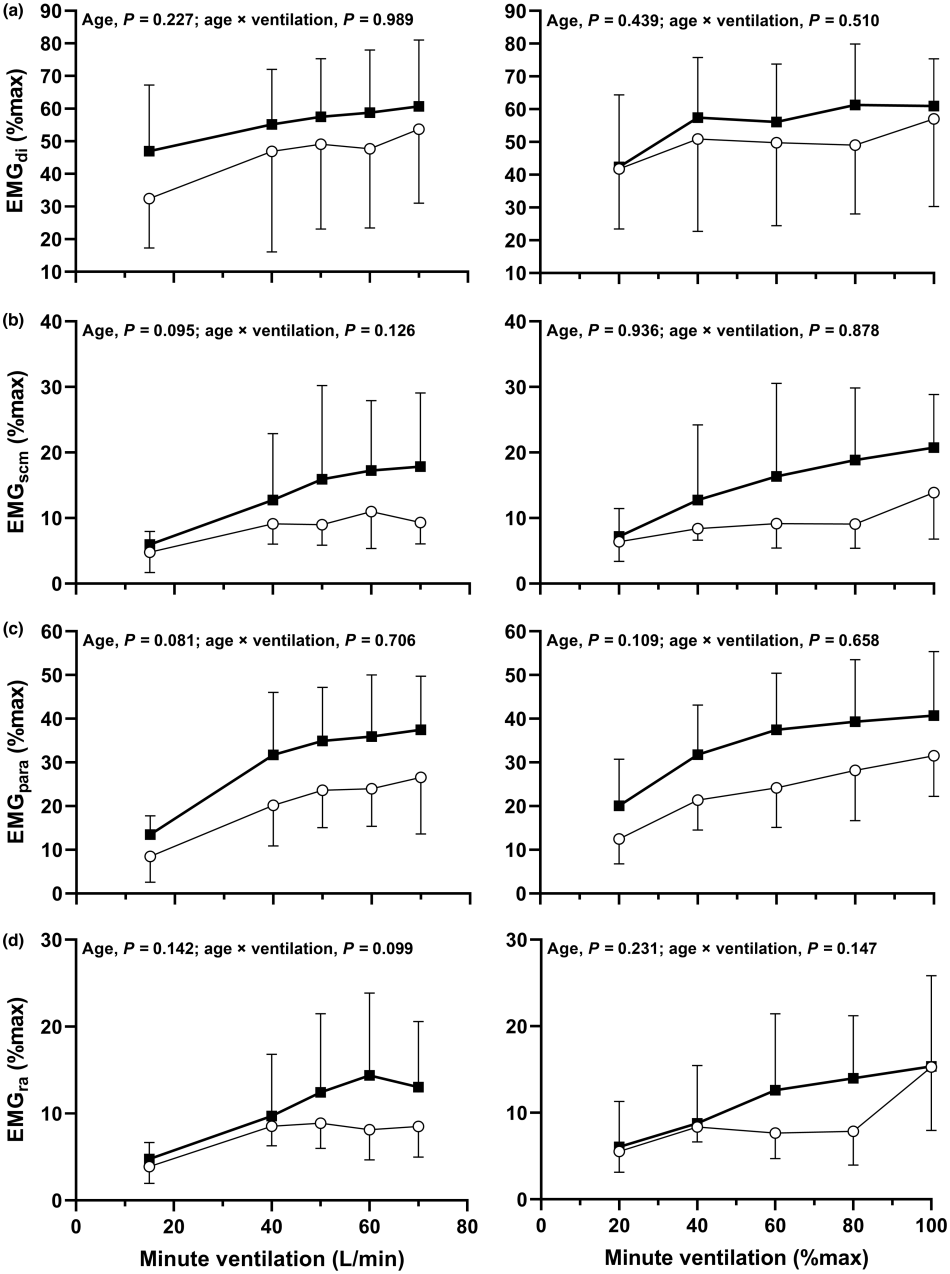
Electromyography (EMG) of the crural diaphragm (EMG_di_; a), sternocleidomastoids (EMG_scm_; b), parasternals (EMG_para_; c), and rectus abdominis (EMG_ra_; d) at rest and during maximal incremental exercise. Responses at absolute (left hand panels) and relative (right hand panels) minute ventilation are shown for younger (open circles) and older men (filled boxes). Panels b‐d have a sample size of *n* = 7 for younger men. Data are presented as mean ± SD. Main effects of age and age × ventilation interaction effects are provided in each panel.

EMG responses at rest and during exercise for the younger and older men are shown in Figure 4, along with main effects of age and age × ventilation interaction effects. EMG_di_, EMG_scm_, EMG_para_ and EMG_ra_ measurements increased during exercise (main effects of absolute and relative ${\dot{V}}_{E}$, *p* < 0.01). No main effects of age or age × ventilation interaction effects were observed, indicating neuromuscular activation patterns in response to exercise were similar in both age groups.

### 3.5 Perceptual responses

**FIGURE 5:**
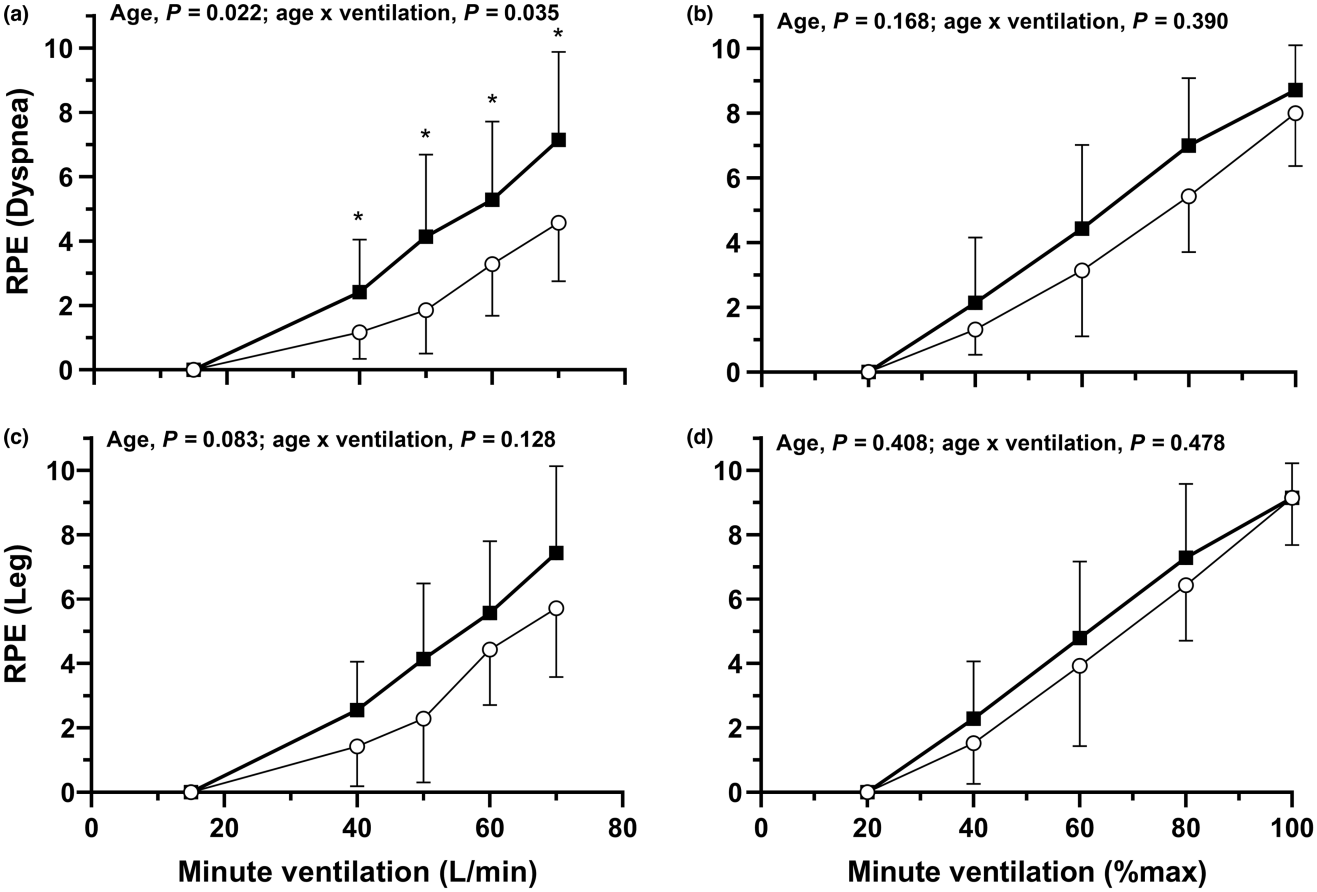
Rating of perceived exertion (RPE) for dyspnea (A,B) and leg discomfort (C,D) at rest and during maximal incremental exercise. Responses at absolute (A,C) and relative (B,D) minute ventilations are shown for younger (open circles) and older men (filled boxes). Data are presented as means ± SD. Main effects of age and age × ventilation interaction effects are provided in each panel.

RPE for dyspnea and leg discomfort at rest and during exercise are shown in Figure 5 with main effects of age and age × ventilation interaction effects. RPE for dyspnea and leg discomfort increased during exercise (main effects of absolute and relative ${\dot{V}}_{E}$, *p* < 0.001). There were no differences between older and younger men for leg discomfort at absolute and relative ${\dot{V}}_{E}$. Older men reported greater dyspnea than younger men during exercise at absolute ${\dot{V}}_{E}$, but no differences were present for dyspnea for relative ${\dot{V}}_{E}$.

**FIGURE 6:**
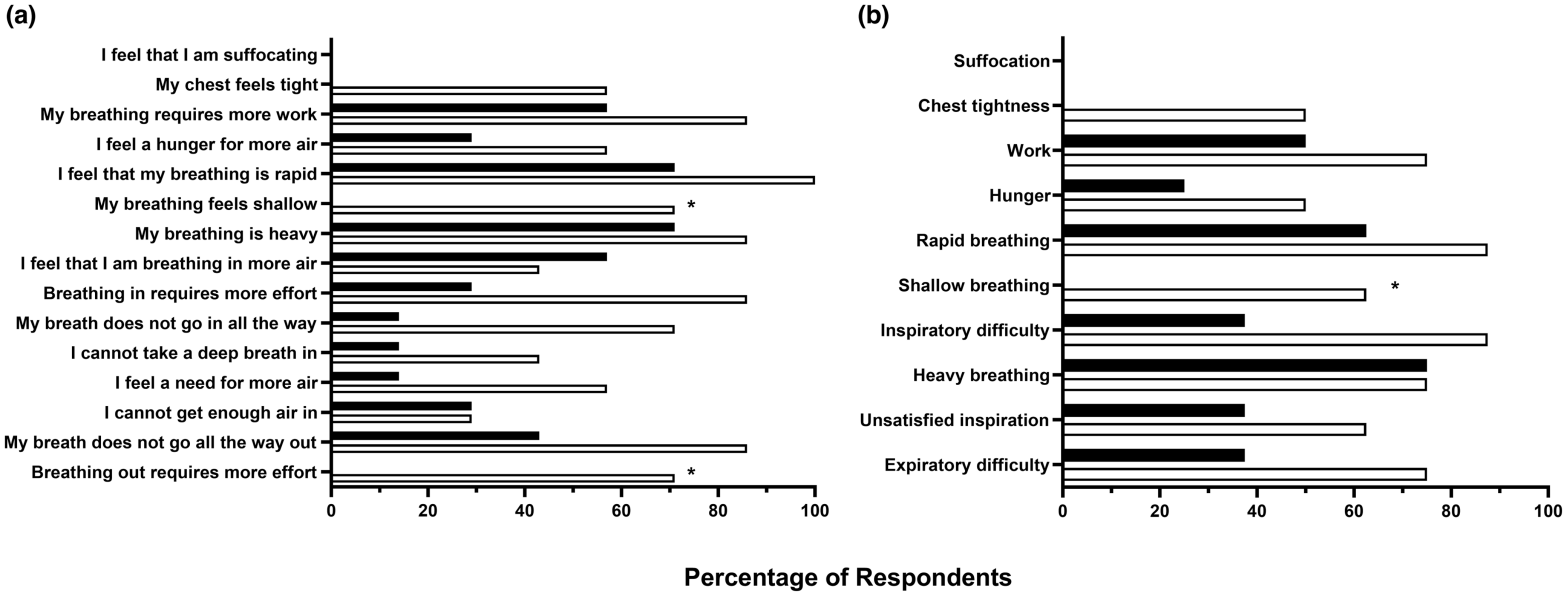
Selection frequency of dyspnea descriptors (a) and dyspnea clusters (b) following maximal incremental exercise. Data are shown for younger (open) and older men (filled). *Significantly different between younger and older men (*p* < 0.05).

Subjective descriptors at the cessation of exercise for the younger and older men are shown in Figure 6. Compared to older men, a greater proportion of younger men reported that “My breathing feels shallow” (*n* = 5 vs. *n* = 0; *p* = 0.021) and “Breathing out requires more effort” (*n* = 5 vs. *n* = 0; p = 0.021). When results were assessed as descriptor clusters, younger men were more likely to report shallow breathing (*n* = 6 vs. *n* = 0; *p* = 0.005) than older men.

## 4 DISCUSSION

### 4.1 Main findings

The aim of this study was to compare respiratory muscle pressure generation and inspiratory and expiratory neuromuscular recruitment patterns between younger and older men during exercise, alongside descriptors of dyspnea. We hypothesized that older men would have greater expiratory muscle recruitment and, an elevated expiratory muscle pressure generation during exercise and select descriptors of dyspnea consistent with an increased respiratory muscle pressure generation following exercise. The main findings were twofold. First, older men demonstrated greater abdominal muscle pressure generation during exercise compared to younger men. Second, older men reported expiratory difficulty and shallow breathing less often than younger men.

### 4.2 Respiratory pressure generation

We observed at rest that both younger and older men had similar abdominal muscle pressure generation with no age‐based differences present in *P*_ga_ or its derived measurements. However, from relatively low intensity exercise (≤50 L/min or ≤ 60% ${\dot{V}}_{E}$) until the limit of exercise tolerance, *P*_ga_ at end‐expiration and *P*_ga_ swing (at absolute and relative ${\dot{V}}_{E}$), and PTP_ga_ (at relative ${\dot{V}}_{E}$) measurements were higher for older compared to younger men. Smith et al. (2018) reported that the expiratory resistive WOB was greater for older compared to younger men during maximal incremental exercise. Our data shows higher *P*_ga_ and PTP_ga_ which are indicative of a higher expiratory WOB. Our data suggest that older men may be utilizing the abdominal musculature to support expiration by compensating for age‐based increases in airway resistance and decreases in chest wall compliance. However, Campbell diagram analysis or more descriptive measures of mechanical ventilatory work would be required to confirm this observation.

Age × ventilation interactions were observed for *P*_es_ at end‐expiration, *P*_di_ swing, and *P*_di_ swing as a percentage of maximum, with higher values for older men than younger men at absolute ${\dot{V}}_{E}$. Despite this, no differences in PTP_es_ or PTP_di_ were observed in older compared to younger men. This finding is in partial agreement with those of Molgat‐Seon et al. (2018b) who reported no differences in PTP_es_ or PTP_di_ at relative ${\dot{V}}_{E}$ between younger and older adults, with differences between the ages only observed at absolute ${\dot{V}}_{E}$. The lack of difference in PTP_es_ or PTP_di_ at absolute ${\dot{V}}_{E}$ may be due in part to the lower $\dot{V}$O_2max_ of participants in the current study compared to those in the Molgat‐Seon et al. (2018b) study. Individuals with a higher $\dot{V}$O_2max_ may utilize the diaphragm and accessory muscles to a greater extent than untrained individuals because these muscles are trained (Erail et al., 2022) to support higher ventilatory work, and this difference may be more apparent during aging.

### 4.3 Respiratory neuromuscular activation patterns

As ventilatory demand increases during exercise, inspiratory and expiratory muscle recruitment is gradually increased to support the increased *F*_*b*_ (Aliverti et al., 1997; Romer & Polkey, 2008). However, little research has been undertaken to understand the impact of healthy aging on respiratory neuromuscular activation patterns. Abraham et al. (2002) measured EMG_ra_ using fine wire intramuscular EMG during incremental and constant load cycling exercise and showed that younger men activated the rectus abdominis early in exercise and that muscle activation plateaued at approximately 20–40% of peak exercise. We also observed an increase in EMG_ra_ during exercise, though no differences were observed between age groups. Why there were no differences in EMG_ra_, yet greater expiratory pressure generation in older men is not clear. It is possible that the rectus abdominis may contribute to postural control during exercise; however, studies in young men have shown that the rectus abdominis is primarily activated for respiration as opposed to postural or locomotive needs (Abraham et al., 2002). Another possibility is differences in older and younger men's mechanical advantage and abdominal musculature neuromuscular activation patterns. As the lung inflates during inspiration from functional residual capacity to total lung capacity, the rectus abdominis and external obliques lengthen by 1–3%, the internal obliques by 15%, and the transversus abdominis by 25% (De Troyer and Boriek, 2011). Furthermore, ventilation induced by inhalation of carbon dioxide enriched gas mixtures in humans activates the transversus abdominis and internal obliques before the rectus abdominis and external obliques (De Troyer and Boriek, 2011). Thus, increases in *P*_ga_ may be predominantly generated by the transversus abdominis and/or the internal obliques rather than the rectus abdominis.

We observed similar inspiratory muscle activation patterns for EMG_di_, EMG_scm_ and EMG_para_ between older and younger men at absolute and relative ${\dot{V}}_{E}$. Our finding contrasts Molgat‐Seon et al. (2018b) who reported that EMG_scm_ was higher at absolute ${\dot{V}}_{E}$ and EMG_di_ higher at absolute and relative ${\dot{V}}_{E},$ in older versus younger participants (men and women). It is possible that our results differ because women were not successfully recruited in our study, particularly given the sex differences in lung function and structure.

### 4.4 Perceptual responses

We observed no differences between older and younger men for leg discomfort at absolute and relative ${\dot{V}}_{E}$. Older men had higher dyspnea ratings than younger men at absolute ${\dot{V}}_{E}$. However, at relative ${\dot{V}}_{E}$ there were no dyspnea differences between the older and younger men. This agrees with other results reporting higher dyspnea ratings during submaximal exercise in older men compared to their younger counterparts (Ofir et al., 2008), but conflicts with others that found no difference between young and old men (Faisal et al., 2015). There were several differences in self‐reported dyspnea descriptors between the age groups. Younger men were more likely to indicate that they were experiencing shallow breathing and expiratory difficulty than older men immediately following exercise. This is despite a lack of differences in their relative and absolute tidal volume or breathing frequency. Ofir et al. (2008) found that shallow breathing was reported by a greater proportion of older men, while Faisal et al. (2015) found no differences in dyspnea descriptors between younger and older adults. Our younger participants' perceptual results thus appear to contradict their physiological data highlighting the dichotomy between the biological and psychological influences of dyspnea perception.

An objective analysis of physiological data thus indicates that younger men's experience of dyspnea should be similar to that of the older men, yet their subjective experience was more unpleasant. The intensity of dyspnea during exercise is reduced by a recent experience of heightened dyspnea (Chang et al., 2023) and that exercising in room air, but believing that air to be hypoxic, results in greater dyspnea (Kipp et al., 2022). These results indicate that desensitization and expectation may play a role in the experience of dyspnea during exercise. It is also possible that desensitization and expectation could impact other aspects of the experience of dyspnea. We therefore suggest that differences in reporting of shallow breathing between our younger and older participants could be due to older participants being more habituated to the feeling of dyspnea and leg discomfort during exercise than our less active younger participants (Sucec et al., 2019). Alternatively, it could indicate that younger and older men undertaking exercise at similar intensities have different subjective experiences of exercise intensity. However, additional studies would be required to confirm either explanation.

Given that we observed higher expiratory muscle pressure generation during exercise in older men, it is natural to consider whether abdominal muscle fatigue may occur. In younger men, abdominal muscle fatigue can be induced by high‐intensity constant load exercise (Hardy et al., 2021; Taylor et al., 2006; Taylor et al., 2013; Taylor & Romer, 2008; Verges et al., 2006), but whether this also occurs in healthy older men is unknown. Abdominal muscle fatigue could have implications for dyspnea, exercise tolerance, and diaphragm fatigue.

### 4.5 Limitations

The main limitation to this study is its sample size which raises the possibility of type II errors. This occurred due to challenges in recruitment, primarily due to the time commitments, perceived invasiveness of testing, and restrictions on human testing due to COVID‐19. Despite our original study design including men and women, only physically active older men were recruited. Though several older women expressed an interest to participate, most were excluded based on health risk factors during screening, and of those accepted into the study (*n* = 4) a significant portion withdrew following falls at home (*n* = 2) or after citing personal reasons (*n* = 1). Younger women perceived the esophageal catheter as too invasive, resulting in very limited recruitment. Sample size was determined from power calculations based upon *P*_ga_ swings during exercise, potentially resulting in underpowered comparisons of measurements such as dyspnea and respiratory neuromuscular activation. Other limitations include the potential for surface EMG recordings to be influenced by “cross talk” from nearby muscle groups. For instance, Abraham et al. (2002) reported that fine wire EMG_ra_ versus surface EMG_ra_ contained lower signal to noise, less artifacts, and recorded a wider range of motor unit sizes. Surface EMG_para_ has also been reported to be a poor surrogate for actual muscle activity compared to fine wire EMG_para_ (Tagliabue et al., 2021), although this conclusion has been questioned (Suh et al., 2021). In addition, EMG_para_ increases when participants cycle with their hands on the ergometer handlebars, likely due to coactivation of the pectoralis muscles (Ramsook et al., 2017). Similarly, EMG_scm_ may be influenced by activation of the scalenes (Mitchell et al., 2018). As the activation of confounding muscle groups cannot be eliminated, we instead minimized their influence by adhering to procedures designed to optimize signal quality. This included skin preparation, participant positioning during exercise, and instructions to minimize excess motion. Future research may benefit from the utilization of fine wire EMG due to its potential to limit cross talk. Surface EMG recordings were normalized against a maximal level of activity achieved during a maximal inspiratory or expiratory pressure, forced vital, or inspiratory capacity maneuver, to limit their susceptibility to interindividual artifacts. EMG_di_ was only measured from the crural, and not the costal, diaphragm during exercise. Thus, our EMG_di_ data does not represent the activation patterns of the diaphragm's entire musculature. Finally, *P*_ga_ may not fully capture the work completed by the contributions of the four muscle groups of the abdominal wall and is thus only an estimate of abdominal wall musculature's workload.

## 5 CONCLUSION

We compared respiratory muscle pressure generation and inspiratory and expiratory neuromuscular recruitment patterns between younger and older men during exercise, alongside descriptors of dyspnea. Older men demonstrated greater expiratory muscle pressure generation during exercise compared to younger men and similar respiratory neuromuscular activation patterns for the diaphragm, sternocleidomastoids, parasternal intercostals and rectus abdominis. We also observed that younger men were more likely to indicate that they experienced shallow breathing than older men during exercise. Elevated expiratory muscle pressures may be due to compensatory mechanisms designed to offset increases in airway resistance due to aging. Future research is required to determine whether older men develop abdominal muscle fatigue in response to constant load high intensity exercise.

## AUTHOR CONTRIBUTIONS

**William MacAskill:** conceptualization, methodology, software, validation, formal analysis, investigation, data curation, writing—original draft, visualization. **Ben Hoffman:** conceptualization, methodology, validation, formal analysis, writing—review and editing. **Michael A. Johnson:** formal analysis, methodology, writing—review and editing. **Graham R. Sharpe:** methodology, writing—review and editing. **Joshua Rands:** investigation, data curation, writing—review and editing. **Shoena E. Wotherspoon:** writing—review and editing formal analysis. **Yaroslav Gevorkov:** methodology, formal analysis, writing—review and editing. **Tracy L Kolbe‐Alexander:** conceptualization, methodology, formal analysis, investigation, resources writing—review and editing supervision (equal). **Dean E. Mills:** conceptualization, methodology, software, validation, formal analysis, resources data curation, writing—review and editing, visualization, supervision (equal).

## FUNDING INFORMATION

University of Southern Queensland. Australian Government Research Training.

Program Scholarship.

## CONFLICT OF INTEREST STATEMENT

The authors declare no conflict of interest.

## 6 ETHICS STATEMENT

The study was approved by the University of Southern Queensland's Human Research Ethics Committee and all procedures conformed to the standards set by the Declaration of Helsinki, except for registration in a database (H17REA261).
